# Supplementary material for: Hierarchical Tactile Sensation Integration from Prosthetic Fingertips Enables Multi-Texture Surface Recognition
Source: Sensors (Basel). 2021 Jun 24;21(13):4324. doi: 10.3390/s21134324 (PMC8271906; doi:10.3390/s21134324)
Supplement: Supplementary file 1 [file sensors-21-04324-s001.zip › Supplementary_LMS_3.pdf]

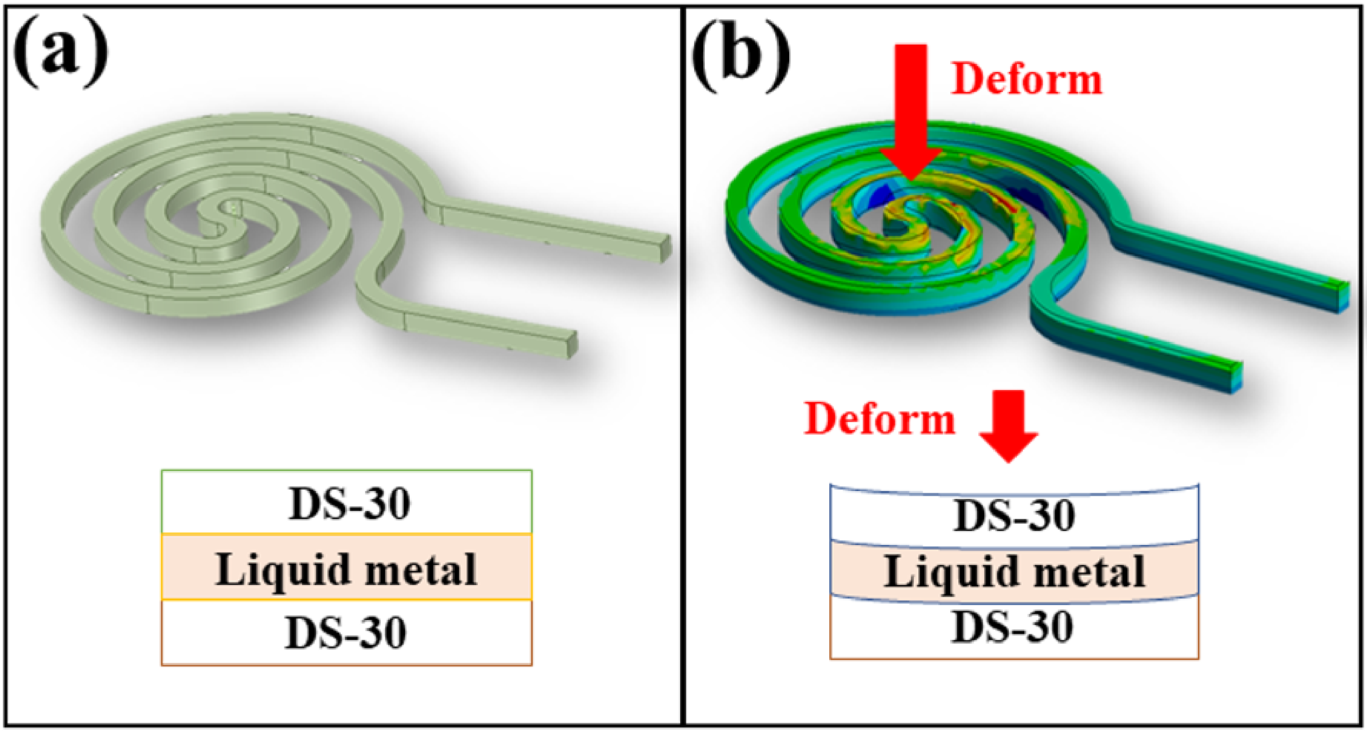

Figure S1. Sensing principle of the LMS. (a) The liquid metal sensor is encapsulated in Dragon Skin-30 (DS-30). (b) Pressing onto the LMS causes deformation of the microchannel containing the liquid metal. This change in cross-sectional area and length of the microchannel changes the electrical resistance of the conductive liquid metal element.

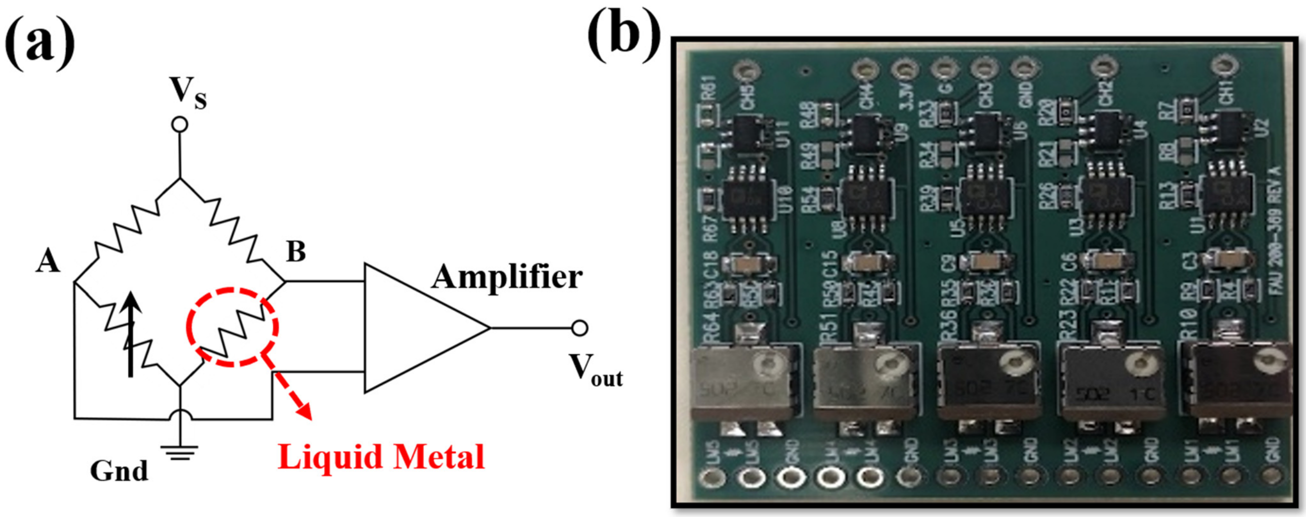

Figure S2. Electronics for the LMS. (a) The liquid metal conductive element was placed in a Wheatstone bridge configuration. (b) A five channel Wheatstone bridge PCB was used to amplify the signals from the LMSs on the fingertips of the i-limb prosthetic hand.

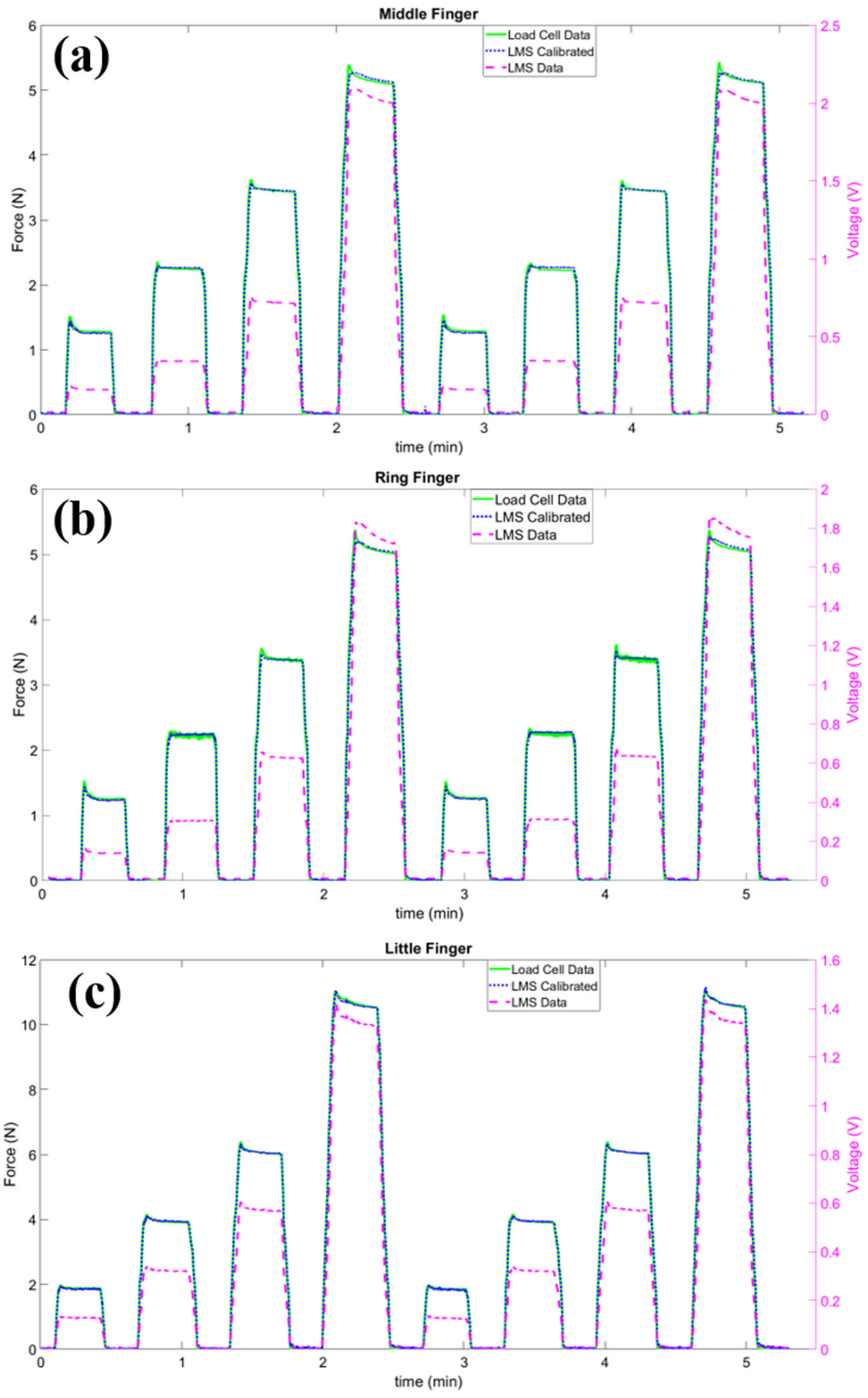

Figure S3. Calibration of the liquid metal sensors. (a) Middle finger calibration. (b) Ring finger calibration. (c) Little finger calibration. An external load cell was used as a reference.

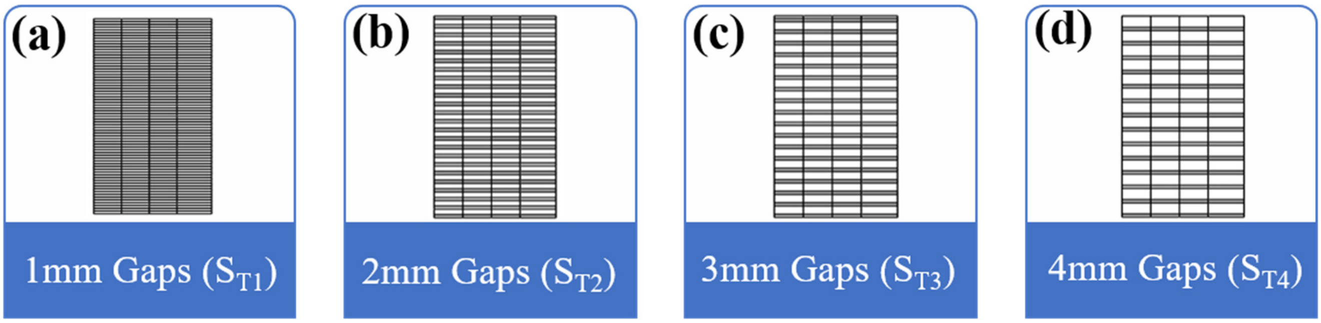

Figure S4. Single-texture surfaces to detect speed of sliding contact and textures. Each of the four different surfaces had four copies of the same texture. (a) Surface S_{T1} had four copies of texture T1 for all four fingers to contact. (b) Surface S_{T2} had four copies of texture T2. (c) Surface S_{T3} had four copies of texture T3. (d) Surface S_{T4} had four copies of texture T4.

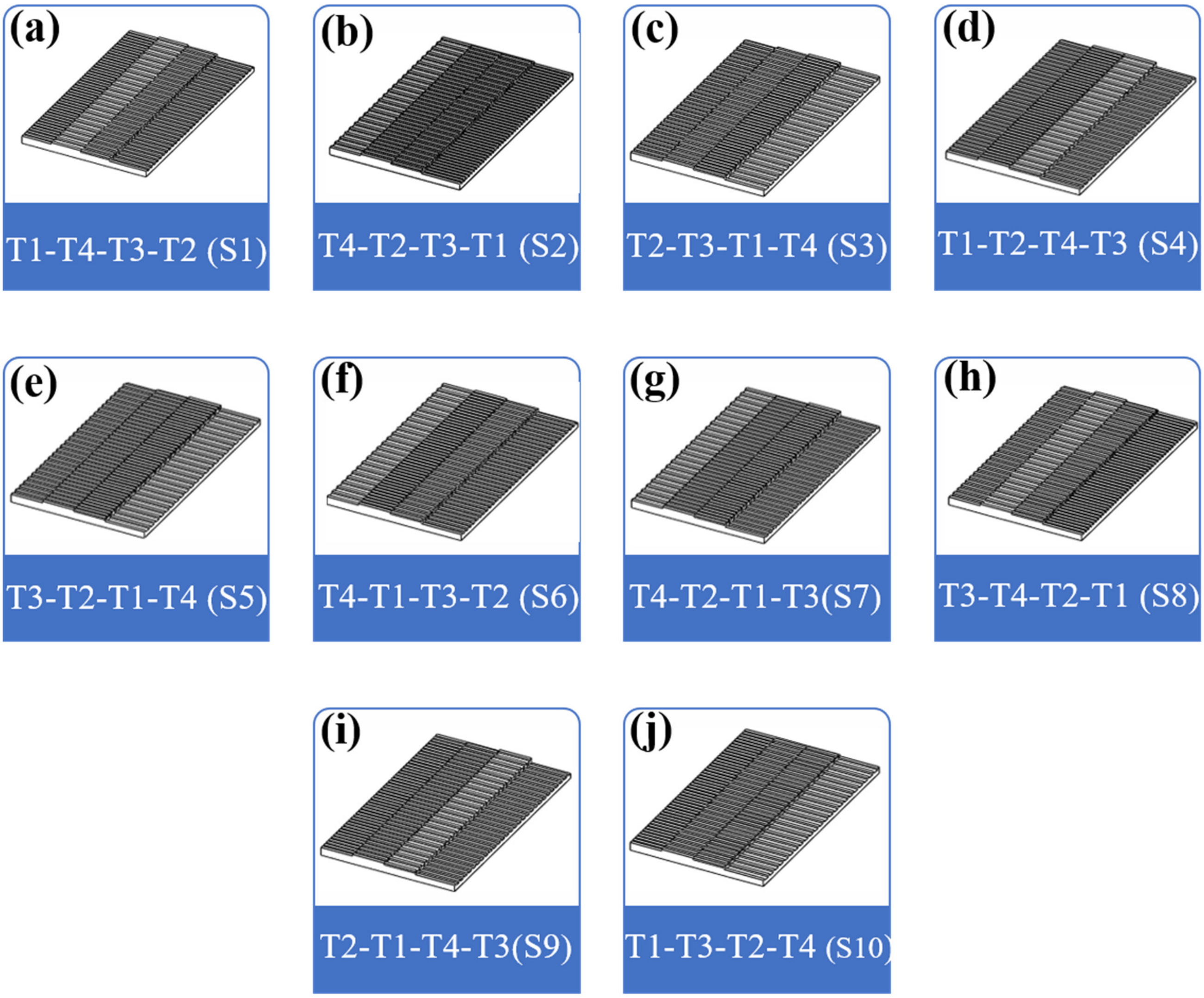

Figure S5. Ten different multi-textured surfaces (S1- S10) were used to validate the potential for hierarchical tactile sensation integration from four fingertips simultaneously. Each of the ten surfaces had the four different textures (T1-T4) in different locations.

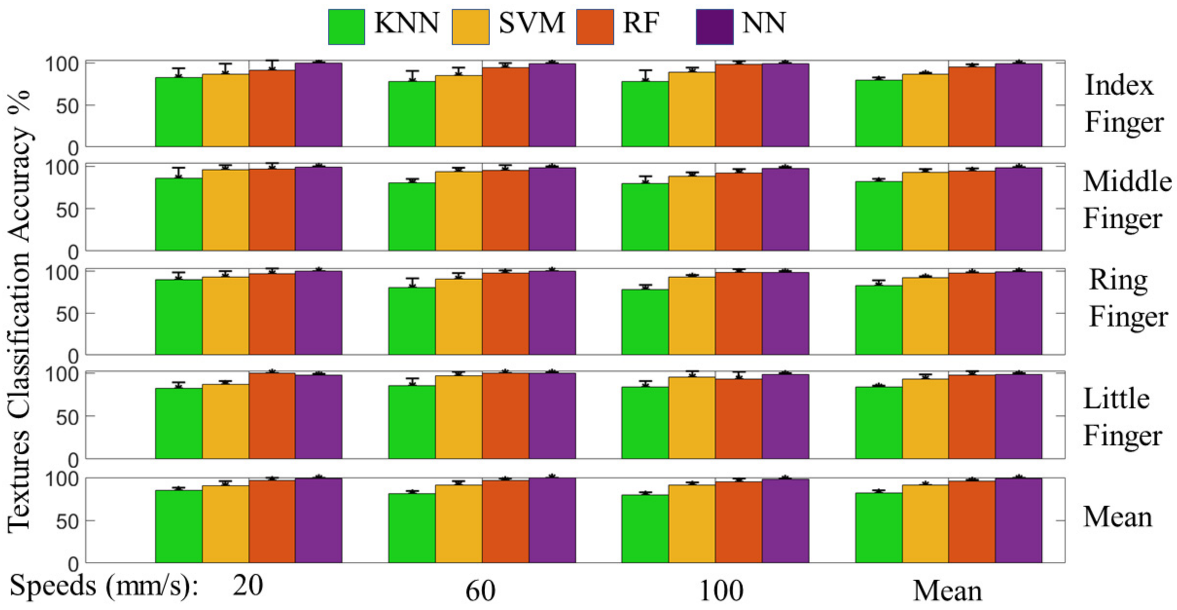

Figure S6. Texture classification accuracy for each finger with three different speeds of sliding contact. See also Figure 9.

Figure S1: Sensing principle of the LMS.
Figure S2: Electronics for the LMS.
Figure S3: Calibration of the liquid metal sensors.
Figure S4: Single-texture surfaces to detect speed of sliding contact and textures.
Figure S5: Ten different multi-textured surfaces (S1- S10).
Figure S6: Texture classification accuracy for each finger with three different speeds of sliding contact.
